# Pressure Stockings for Edematous Feet in the Lepra Reaction: An Inexpensive Patronage

**DOI:** 10.7759/cureus.58893

**Published:** 2024-04-24

**Authors:** Ghazal Ahmed

**Affiliations:** 1 Dermatology, Venereology and Leprosy, All India Institute of Medical Sciences, Deoghar, Deoghar, IND

**Keywords:** lepra reactions, compression stockings, steroid, hensen’s disease, leprosy

## Abstract

Leprosy has been known for its wide range of peripheral nerve and tissue involvement and causing disabilities. Early diagnosis and treatment with multi-drug therapy can save lives and limbs and prevent disabilities. However, management and drug therapy are usually lengthy and full of ups and downs of side effects. Further, the lepra reaction is frequently noted during management, requiring immunosuppression and leading to associated side effects. Limb edema per se due to leprosy is unusual and mostly a symptom of a reactional state. There is no specific management for edema in such cases, and it subsides with improving reactionary states. Nevertheless, the edema may be persistent and bothersome. The present report highlights one such unusual case in a 40-year-old man, diagnosed with borderline-tuberculoid leprosy and experiencing a type-1 reaction. Owing to ocular complications, steroid therapy for the reaction was tapered abruptly, and his limb edema did not subside with the improving lepra reaction. Compression stockings helped to manage edema. This case also makes us ponder the possible use of compression stockings as an opioid-sparing aid in lepra reaction-related edema.

## Introduction

Skin involvement in leprosy is frequent, but edema per se due to leprosy is unusual and mostly a symptom of a reactional state [1]. There are reports of patients presenting with edema and dactylitis as the presenting symptoms of lepra reactions and leprosy [2,3]. Such tenosynovitis and edema are reported in the hands and joints. Although the exact mechanism is yet to be established, direct involvement by *Mycobacterium leprae*, complement activation, and immune complex deposition in lepra reactions are thought to be pathophysiological genesis [4]. Lepra reactions lead to inflammatory changes due to circulating immune complexes. On the other hand, they can cause vasculitis and tissue edema, which may in turn obstruct lymph flow. Vasculitis, tissue inflammation, and lymphy flow obstruction might cause edema in such patients. Management usually involves addressing lepra reactions, which are managed using immunosuppressants like steroids, continued multi-drug therapy (MDT) for leprosy, and other symptomatic supportive measures [5]. The use of supportive stockings to aid edema management and their probable steroid-sparing role has yet to be discussed.

## Case presentation

**Figure 1:**
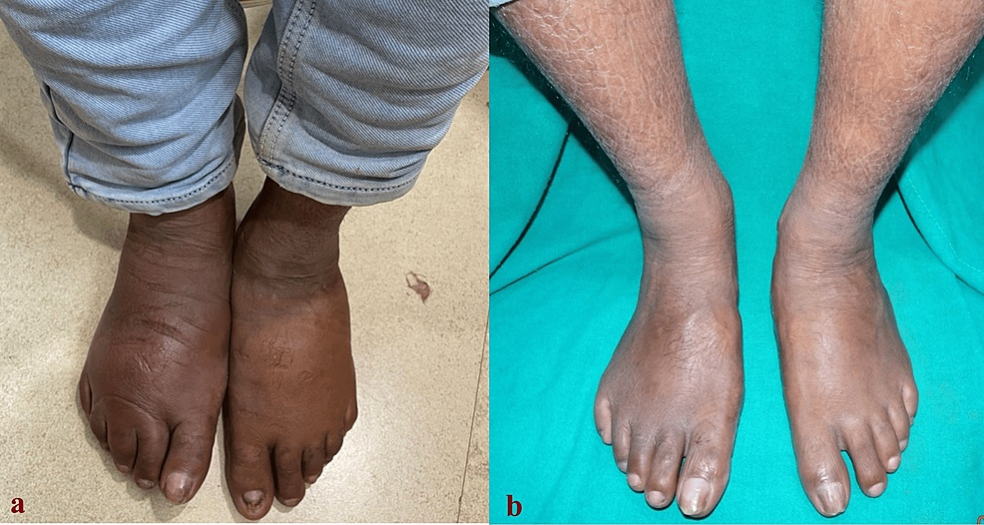
(a) Feet edema before and (b) follow-up at seven months of using pressure stockings. The follow-up image shows visible veins, indicating the absence of edema

A 40-year-old man with an average build was diagnosed with borderline-tuberculoid leprosy with type-1 reaction and was started on MDT using rifampicin, dapsone, and oral steroids. The patient was doing well, and his reaction was under control. However, he developed eye pain, tearing, and slightly blurry vision. Intraocular pressure measurement showed borderline pressure, and complications from systemic steroids were considered. Thus, the steroid was tapered fast and stopped. His reaction-related signs and symptoms improved, but his feet swelled due to edema, which was distressing for the patient. The edema was pitting in nature and predominantly noted below the knee. Initially, it was thought to be the reappearance of a type-1 reaction. However, examination of his truncal patches showed that they were minimally inflamed and in the process of resolving. His cardiovascular, renal, and abdominal examinations were unremarkable. Thyroid function tests, renal function tests, and serum electrolytes remained within normal ranges during follow-up, and even the liver function test results did not indicate failure. Hence, a conservative management approach was decided; pressure stockings were advised for the edematous feet, and MDT was continued without any steroids or other immunosuppressants. It worked well for the patient. He completed his MDT, and his foot edema improved. The pre-treatment and follow-up at seven months of using pressure stockings images are shown in Figures 1a-1b, respectively.

The limb veins were visible, indicating the absence of edema. He was relieved from treatment but was advised to continue using pressure stockings as lifestyle support.

## Discussion

Usually, edema in the hands and feet was thought to be a part of type-1 lepra reaction, primarily when there is associated erythema and edema of the leprosy patches elsewhere in the body, and the patient is treated in the line of lepra reaction with immunosuppressants [6]. While it's important to remember that acral edema is a feature of leprosy per se, as well due to the compromise in the autonomic nervous system, in our case, foot edema might have been part of the reappearing lepra-1 reaction, or even due to the fast tapering of steroids [7]. Reports of edema following leprosy-related arthritis are described [8]. However, our patient did not have any joint-related complaints or clinical findings to indicate so.

Nevertheless, whatever the cause of edema, it is encouraging to see that the simple use of pressure stockings helped to avoid systemic steroids and other immunosuppressants. The mechanism behind the effect can be explained by the increased flow of trapped lymph through the lymph vessels caused by the compression of muscles, which acts as an extra force. This approach can help us in many ways: total avoidance of steroids or enabling us to use them in a lower dose or shorter duration if lepra-1 reaction manifests with edematous feet. It also helps to prevent trophic ulcers and is being reported to be used for disability prevention by the Pan American Health Organization in their guidelines for primary health care services [9]. Compression stockings have been advised for occupational leg edema [10]. In a report, de Godoy et al. indicated using compression stockings to maintain the limb circumference following lymphedema of filariasis [11]. Pitting edema is usually caused by heart failure, liver failure, renal failure, and early-stage hypothyroidism. Our patient's clinical and laboratory examinations found no abnormalities for these systems, indicating that the edema was most likely due to a lepra reaction. While edema following a lepra reaction is well known, literature describing the use of pressure stockings for managing such edema is either unavailable or scarce.

## Conclusions

The limb edema followed by a lepra reaction or abrupt steroid discontinuation might be persistent and bothersome. Compression stocking application might aid as supportive therapy. While our experience with the single case cannot build an association with the steroid-sparing effect, it indeed lets us ponder for further observation in this line.
